# De novo genome assembly and annotation of *Gnathostoma spinigerum*

**DOI:** 10.1186/s13071-026-07378-1

**Published:** 2026-04-11

**Authors:** Belén González-Bertolín, Sara Monzón, Ángel Zaballos, Pilar Jiménez, Paron Dekumyoy, Poom Adisakwattana, Sarai Varona, Isabel Cuesta, Sunita B. Sumanam, Javier Sotillo, Ana Hernández-González, Iñigo Marcos-Alcalde, Paulino Gómez-Puertas, Neil D. Young, Maria J. Perteguer

**Affiliations:** 1 Parasitology Reference and Research Laboratory, Helminths Unit, National Centre for Microbiology, Instituto de Salud Carlos III, Majadahonda, Madrid, Spain; 2 Eicosanoids Research Division, Institute of Biomedicine and Molecular Genetics (IBGM), Consejo Superior de Investigaciones Científicas (CSIC), Valladolid, Spain; 3 CIBER de Diabetes y Enfermedades Metabólicas Asociadas (CIBERDEM), Instituto de Salud Carlos III, Madrid, Spain; 4 Bioinformatics Unit, Core Scientific and Technical Units, Instituto de Salud Carlos III, Madrid, Spain; 5 Genomics Unit, Core Scientific and Technical Units, Instituto de Salud Carlos III, Madrid, Spain; 6 Department of Helminthology, Faculty of Tropical Medicine, Mahidol University, Bangkok, Thailand; 7 Department of Veterinary Biosciences, Faculty of Veterinary and Agricultural Sciences, Melbourne Veterinary School, The University of Melbourne, Parkville, VIC Australia; 8 Molecular Modeling Group, Centro de Biologia Molecular Severo Ochoa (CBM, CSIC-UAM), 28049 Madrid, Spain; 9 CIBER de Enfermedades Infecciosas (CIBERINFEC), Institute of Health Carlos III, Madrid, Spain

**Keywords:** *Gnathostoma*, *G. spinigerum*, Genome, De novo assembly, Annotation, M10 metalloproteinases, Tissue inhibitors of metalloproteinases, TIMPs

## Abstract

**Background:**

*Gnathostoma spinigerum* is a parasitic nematode implicated in human cases of eosinophilic meningitis. This species is primarily endemic to Thailand and frequently occurs in other regions as imported cases. Although this parasite poses a significant pathogenic risk, its genome has not yet been assembled or annotated. The aim of our study is to generate the first genome assembly of *G. spinigerum*.

**Methods:**

Whole-genome sequence libraries were generated from genomic DNA extracted from a pooled sample of advanced stage 3 larvae. After sequencing, the assembly of the genome was produced using a combination of second- and third-generation sequencing technologies. Multiple draft assemblies were generated and evaluated, and the absence of contamination was determined. Identification and modeling of new *G. spinigerum* metalloproteinases and tissue inhibitors of metalloproteinases were performed, and molecular dynamics simulations were used to analyze their potential interactions. The final assembly was annotated and made publicly available via the NCBI genome database.

**Results:**

The hybrid assembly approach (using short and long reads) using the SPAdes assembler and with post-assembly polishing (Pilon/Picard) yielded the most complete genome (222-Mb genome size, N50 = 14,149 bp, 69.6% BUSCO assembly). A total of 14,451 protein-coding genes were predicted in the *G. spinigerum* genome with 62.3% BUSCO annotation completeness. Three-dimensional computational modeling and molecular dynamics of six new metalloproteinases and two new tissue inhibitors of metalloproteinases are presented.

**Conclusions:**

We provide the first sequence assembly and annotation for the nematode *G. spinigerum*. This draft genome will be an essential resource for future scientific and applied investigations of diseases caused by this parasite.

**Graphical abstract:**

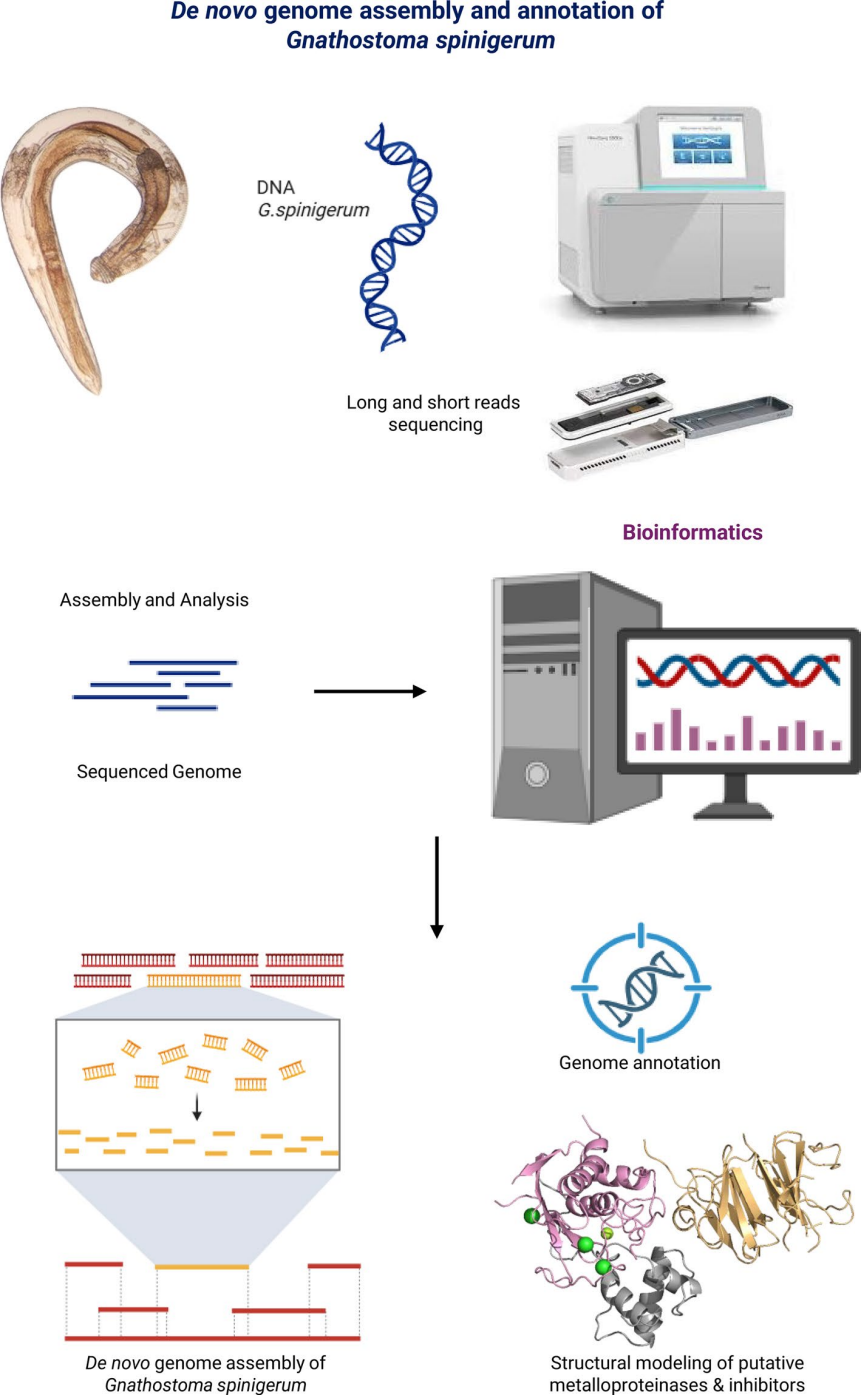

**Supplementary Information:**

The online version contains supplementary material available at 10.1186/s13071-026-07378-1.

## Background

Parasitic infection by *Gnathostoma spinigerum* (gnathostomiasis) can lead to a severe condition of cerebral involvement, potentially triggering eosinophilic meningitis or meningoencephalitis caused by advanced third-stage larvae (L3). Efforts to develop effective diagnostics and therapeutics for gnathostomiasis have been limited, partly by the lack of genomic information available for this parasite.

In 1836, Richard Owen described the genus *Gnathostoma* spp. following the discovery of adult parasites in the stomach of a Bengal tiger (*Panthera tigris*), naming the species *G. spinigerum* [1]. Humans are incidental hosts who become infected mainly by eating raw or undercooked freshwater fish containing L3 larvae [2]. In humans, the larvae are unable to develop into adults and can migrate through various tissues and organs [3, 4]. The most common symptoms are skin-related, but the most severe form of visceral involvement is invasion of the central nervous system (CNS), including the brain and spinal cord, which can lead to death [5]. Members of the genus *Gnathostoma* are distributed worldwide, but *G. spinigerum* is the only species currently linked with cases of cerebral involvement. Gnathostomiasis has been reported in Japan, India, China, and Southeast Asia, including Thailand, Laos, Myanmar, Indonesia, Malaysia, and the Philippines [1]. The common incidence of *G. spinigerum* infection underscores the significant public health concern posed by gnathostomiasis, with a notable concentration of human cases reported in Thailand [6]. Moreover, gnathostomiasis has emerged as a sporadic yet alarming phenomenon in non-endemic regions, fueling concerns about its potential for global dissemination. Travel-related cases have been documented in Europe, Australia, and the Americas, reflecting the disease's ability to transcend geographical boundaries [7, 8].

The absence of expedited therapeutic intervention in gnathostomiasis can result in severe and prolonged morbidity. The parasite, with a longevity in the human host that may exceed a decade, is responsible for asymptomatic intervals followed by symptomatic recurrences. The clinical sequelae are significant, and involvement of the CNS confers a risk of fatal outcome. The treatments of choice (albendazole and/or ivermectin with corticosteroids) do not ensure a complete cure, with several case relapses documented. No definitive mechanisms of resistance to available drugs have been described for *G. spinigerum* so far [9].

A reference genome for *G. spinigerum* will provide a much-needed molecular resource for gnathostomiasis diagnostic and therapeutic advancements. Our study aims to fill this knowledge gap by achieving a draft genome assembly of *G. spinigerum*.

## Methods

### DNA extraction of parasitic larvae

*Gnathostoma spinigerum* larvae were collected from the livers of naturally infected eels (*Monopterus albus*) in Thailand. Briefly, livers of freshwater eels were extracted and digested with 1% hydrochloric acid-pepsin in a 37 °C water bath for 3 h. Larvae were identified by microscopy and washed several times with 85% saline and distilled water. A total of 250 advanced third-stage larvae (L3), corresponding to human infective forms, were kept at − 80 °C.

DNA extraction was conducted, employing automated methodologies (QIAcube), with the QIAamp DNA Mini Kit combined with the ATL buffer (QIAgen) for 48 h at 56 °C.

Each *G. spinigerum* larva was taxonomically verified by sequencing mitochondrial (COI) and ribosomal (ITS) genes using a previously published conventional polymerase chain reaction (PCR) protocol [10]. Both PCR amplicons were sequenced using the Sanger sequencing method on a 3730XL DNA Analyzer (Applied Biosystems).

### Library preparation and sequencing

Two sequencing technologies were employed: Illumina and Oxford Nanopore Technologies (ONT). For Illumina, four samples were prepared, and two different library preparation kits were utilized: the Nextera DNA Flex Library Prep kit (Illumina) with eight amplification cycles, following the manufacturer's instructions. The samples used with this library were ILGs-1 and ILGs-2 (*n* = 10 pooled larvae in each library). Subsequently, the libraries were quantified using the Promega QuantiFluor® ONE dsDNA System, and their average size was determined using the high-sensitivity 2100 Bioanalyzer (Agilent). These first two samples were sequenced in a NextSeq 550 (Illumina), using the sequencing kit NextSeq 500/550 High Output Kit v2.5 (300 cycles) (Illumina). The second sequencing kit employed was DNA PCR-Free Prep, Tagmentation (Illumina), which did not require amplification. The samples used for this second Illumina modality were ILGs-3 and ILGs-4 (*n* = 51 larvae per library). The second set of samples was sequenced using a NovaSeq sequencer (Illumina) with the NovaSeq 6000 SP Reagent Kit v1.5 (300 cycles) (Illumina). In both cases, the reads obtained with the Illumina technology were paired end and 150 bp long.

Before sequencing the long reads, the libraries were prepared using the Ligation Sequencing Kit SQK-LSK110 (Oxford Nanopore Technologies) and the NEBNext Companion Module for Oxford Nanopore Technologies Ligation Sequencing (New England Biolabs), following the manufacturer's instructions. ONTGs-5 sample consisted of *n* = 162 pooled larvae. For the long-read approach, the MinION MK1C (Oxford Nanopore Technologies) sequencer was employed, while the flow cell was (R9.4.1) FLO-MIN106D (Oxford Nanopore Technologies). Basecalling of raw FAST5 files was performed using the Guppy v5.1.12 program, and reads were stored in FASTQ format.

### Genome assembly approaches

All bioinformatics analyses were performed on a high-performance computing (HPC) cluster from the Instituto de Salud Carlos III.

The FastQC software (v.0.11.9) [11] was employed to assess the quality of the FASTQ files, and any necessary read filtering steps were performed using the fastp program (v.0.20.0) [12].

**Fig. 1:**
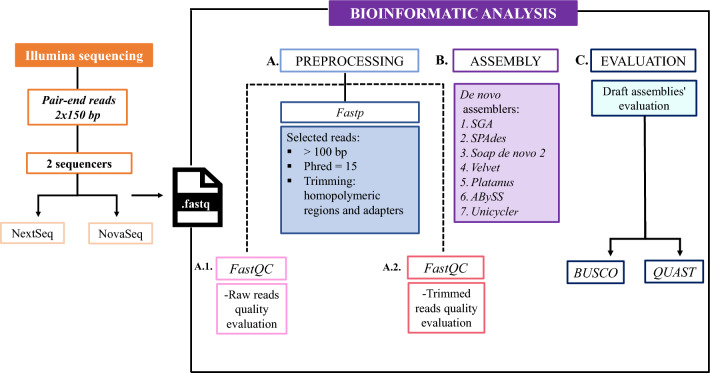
Short-read genome assembly workflow. **A**–**C** Boxes represent the main processes in the short-read bioinformatic analysis, after obtaining fastq files from Illumina sequencing. **A** Preprocessing was performed in three steps: first, FastQC analysis to get the quality evaluation of raw reads (**A.1**); second, Fastp, the main preprocessing step, which filtered the reads that were > 100 bp and those with a Phred score > 15, and finally trimmed homopolymeric and adapter regions (detailed in supplementary material); third, FastQC (**A.2**) again obtained the quality evaluation of selected and trimmed reads. **B** Assembly process using different de novo assemblers. **C** Evaluation process of the several draft assemblies. Each was evaluated using the Benchmarking Universal Single-Copy Orthologs (BUSCO) and Quality Assessment Tool for Genome Assemblies (QUAST) software to obtain metrics to compare and select the best draft assembly

The de novo assembly of the genome was performed individually using: (i) short reads from the Illumina (NextSeq) technology with the following assembler: SGA (v.0.10.15) [13], SPAdes (v.3.15.4) [14], Soap de novo 2 (v.2.04) [15], Platanus (v.1.2.4) [16], ABySS (v.2.3.5) [17], and Unicycler (v.0.5.0) [18]; (ii) short reads from the Illumina (NovaSEQ) technology with SPAdes (v.3.15.4); (iii) long reads from the ONT technology with Flye (v.2.9) [19] and NECAT (v.0.0.1) [20]. The quality evaluation of the different assemblies was performed with QUAST (v. 5.0.2) [21] and BUSCO (v. 5.3.2) in genome mode. [22] The workflow of the short-read approach is summarized in Fig. 1

**Fig. 2:**
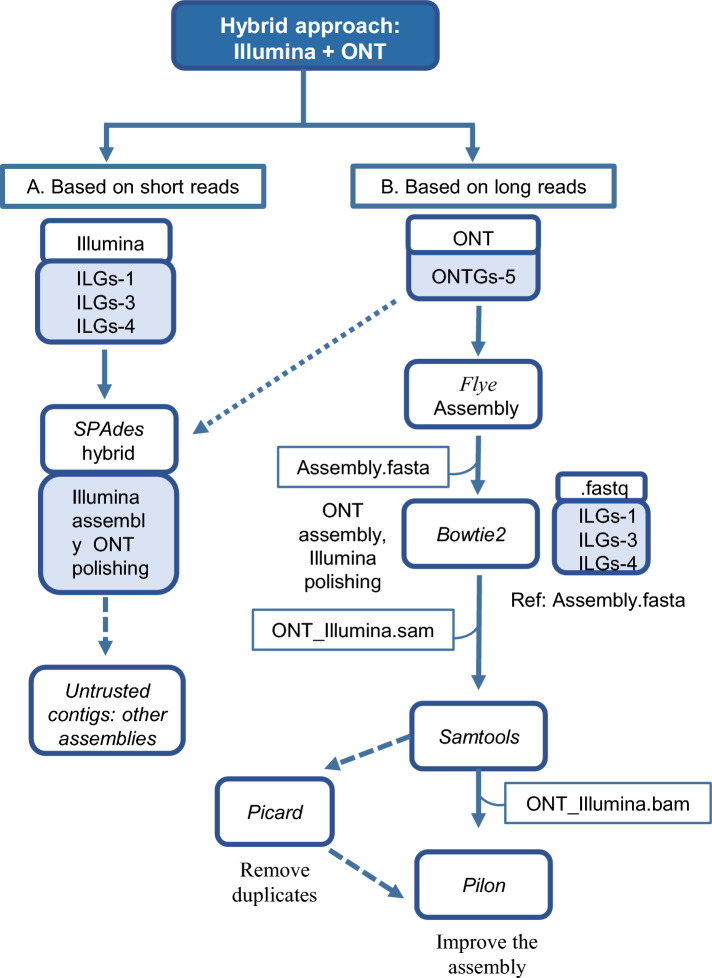
Hybrid genome assembly workflow. **A** Based on short reads, the SPAdes assembler was employed using the hybrid mode, incorporating short (Illumina) and long reads from ONT technology with the nanopore option. **B** Based on long reads, it used contigs assembled using Flye and the long reads to align short reads (Illumina) employing Bowtie2

After obtaining draft genome assemblies for each sequencing technology, hybrid assemblies were constructed. Specifically, short reads were integrated with long reads to create hybrid assemblies. Two distinct approaches were employed for this purpose. A workflow of the whole process is shown in Fig. 2. Two assembly approaches were used: one a hybrid SPAdes assembly of both Illumina and ONT reads and the other a Flye assembly of long reads that was then polished with Illumina reads as follows. Reads were mapped to the genome using Bowtie2 (v.2.4.2) [23]. Alignment files were converted from SAM to BAM format using SAMtools (v.1.12) [24]. Duplicates were removed using the Picard program (v.2.25.1) [25]. The contigs were then corrected using the final BAM, the Flye genome assembly, and Pilon (v.1.23) [26]. The same evaluation procedure was used for the generated draft assemblies with BUSCO and QUAST.

Further information related to the HPC, genome assembly software, and approaches is included in Additional file 1 (text 1).

### Structural and functional annotation

Repeat element annotation was carried out using RepeatModeler (v.2.0.5) [27], RepeatMasker (v. 4.1.6) and the DFAM repeat database [28].

Protein-coding gene annotation of the final draft genome assembly was performed using the Braker pipeline (v3.0.8) [29]. Gene evidence for Braker was obtained by genome mapping of metazoan protein sequences and previously published RNAseq data from *G. spinigerum* (NCBI accession no. SRR8137628) [30]. The selected gene finders were GeneMark-ET and AUGUSTUS, and the gene sets were combined using TSEBRA. Functional annotation of inferred proteins was performed using eggNOG-mapper (v 2.1.12) based on eggNOG data [31] and InterproScan (v. 5.56) [32]. BUSCO (protein mode) and OMArk software [33] (v.0.3.0) was used to measure proteome completeness. The latter software characterizes the consistency of the protein-coding genes after annotation with the genome assembly and transcriptome, also identifying the presence of contamination from other species.

### Mitochondrial genome assembly comparison

A comparison was conducted with the previously published mitochondrial genome of *G. spinigerum* [34] to assess the quality of the assembled genome. This evaluation involved aligning the draft assembly with the best-performing metrics to the corresponding mitochondrial genome sequence using the command-line BLAST tool (v.2.11.0). To identify gene families associated with host-parasite interactions, we performed targeted searches for matrix metalloproteinases (MMPs) and SCP/TAPS proteins. The latter were identified by searching the predicted proteome against the Pfam CAP domain profile (PF00188) using hmmsearch (HMMER v3.4).

### Structural modeling of putative metalloproteinases M10 and tissue inhibitors of metalloproteinases (TIMPs) identified in the genome. Molecular Dynamics simulation of M10-TIMP interaction

Identification of the proteins corresponding to sequences in the *Gnathostoma spinigerum* genome predicted as putative metalloproteinases M10 and/or tissue inhibitors of metalloproteinases (TIMPs) was performed by searching for conserved domains. We performed genome-wide identification of M10 metalloproteinases and TIMPs in the *G. spinigerum* protein-coding gene set using Hidden Markov Models (HMMER v3.3.2) against the Pfam-A database (PF00413 for M10 and PF00965 for TIMPs). Hits with < 60% domain coverage were excluded. Structural modeling of the newly identified metalloproteinases and TIMPS, as well as the only *G. spinigrum* metalloproteinase M10 described so far in Genbank (AAF82802), was performed using as template the crystal structure of the human proMMP-9 catalytic domain (Protein Data Bank id: 7OGT) [35], the human pro-matrix metalloproteinase-2 (Protein Data Bank id: 1CK7) [36], or the human tissue inhibitor of metalloproteinases-2 (Protein Data Bank id: 1BR9) [37]. The structures were modeled by combining residue positions obtained using Phyre 2.2 [38] and SwissModel [39].

The interaction complexes between the seven M10 metalloproteinases and the two TIMPs (in total, 14 models) were obtained using the structure of the complex between the human tissue inhibitor of metalloproteinases-1 and the matrix metalloproteinase-3 catalytic domain (Protein Data Bank id: 6N9D) [40] as a template. Structural complexes were subjected to 200 ns of unrestrained molecular dynamics (MD) simulation using the Amber18 package (https://ambermd.org; University of California-San Francisco, CA), essentially as previously described [41]. Briefly, after solvation, initial wild-type and variant model structures were subjected to 10,000 cycles of energy minimization, followed by a 1-ns restrained equilibration phase in which the temperature was smoothly raised to 297 K, after which the restraints were gradually removed over 10 ns. Each system was then subjected to a 2000ns free MD production phase.

Trajectories were analyzed using cpptraj [42] and VMD [43]. The NAMD Energy Plugging of VMD was used to evaluate the nonbonding energy contributions to the surface interactions between M10 metalloproteinases and TIMPs during the simulations using NAMD [44]. Plots were generated using Pymol (https://pymol.org).

Detailed arguments for programs described in this section are given in Additional file 1, Text 1.

## Results

### Microscopical and molecular classification of *Gnathostoma* larvae

**Fig. 3:**
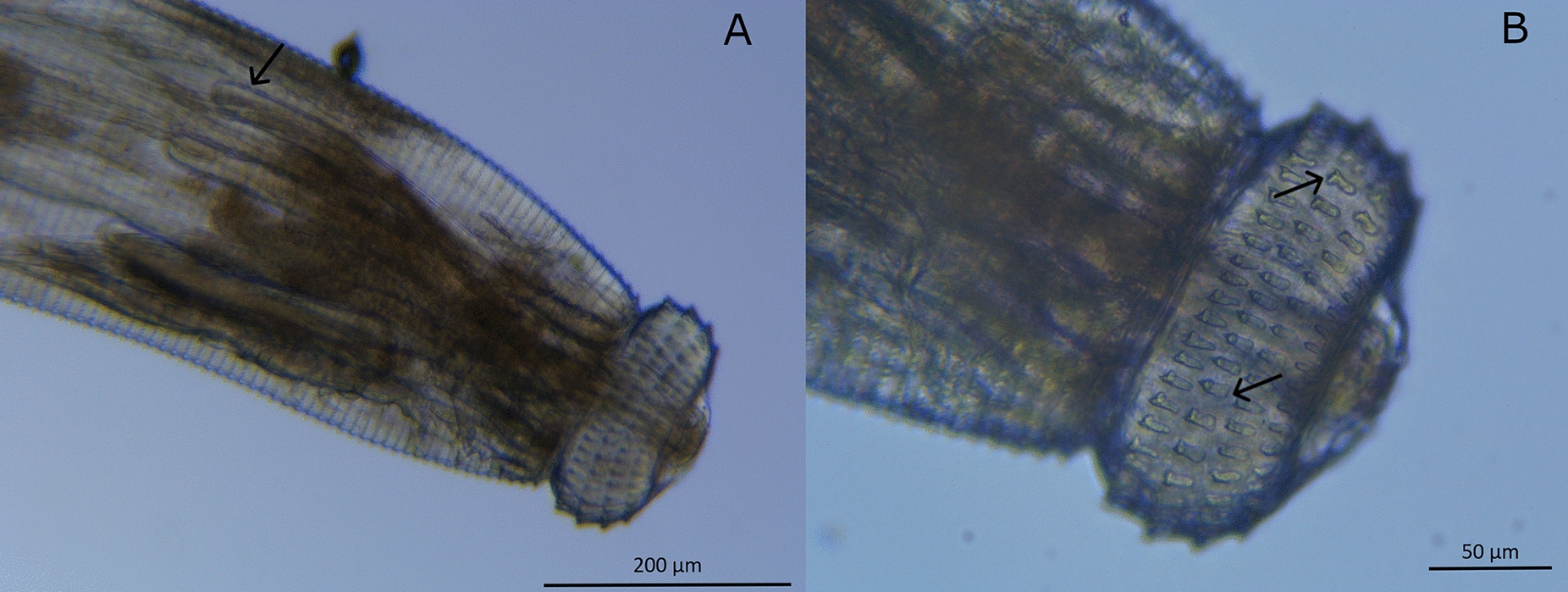
**A** Larva of *Gnathostoma spinigerum* advanced stage 3. Cervical sacs (black arrows) and cephalic bulb. **B** Magnification of the head bulb, with four rows of hooklets and lips, details (black arrows) of the hooklets

Microscopic observation confirmed that the advanced 3 larvae (AL3) belonged to the *Gnathostoma spinigerum* species (Fig. 3). The *G. spinigerum* characteristics follow the criteria of a cephalic bulb bearing four rows of hooklets, a long muscular esophagus, and four cervical sacs (Fig. 3A). A higher magnification of the anterior end of the body is displayed in Fig. 3B with the two lips located at the extreme. The cephalic bulb hooklets have an oblong shape and a sharp-pointed end at the base. Each row has ≥ 40 hooklets.

The ribosomal ITS2 and mitochondrial (COI) PCR amplified DNA products were 650 and 250 bp, respectively. Upon sequencing, the BLAST results indicated 100% identity with the species *G. spinigerum* (GenBank accession no. MK033974.1).

### DNA extraction and library evaluation

The DNA concentrations of the different samples after extraction were as follows: 0.539 ng/µl (ILGs-1), 0.882 ng/µl (ILGs-2), 0.7 ng/µl (ILGs-3), 2.4 ng/µl (ILGs-4), and 5.5 ng/µl (ONTGs-5). After construction of the short-read libraries, the concentrations were 9.5 ng/µl (ILGs-1), 2.8 ng/µl (ILGs-2), 0.243 ng/µl (ILGs-3), and 0.975 ng/µl (ILGs-4). For the long-read sample (ONTGs-5), 258.5 ng of genomic DNA was used to make the library.

### Sequencing and pre-processing

Illumina short-read DNA libraries were constructed from four of the five samples processed, resulting in the sequencing of paired-end 2 × 150-bp reads for samples ILGs-1, ILGs-2, ILGs-3, and ILGs-4. Raw and filtered reads for each library, including the platform used to sequence the data, are detailed in the supplementary material (Table S1).

The Illumina-NextSeq sequenced ILGs-1 and ILGs-2 samples had a guanine-cytosine content of 37%. Samples ILGs-3 and ILGs-4, sequenced using Illumina NovaSeq technology, yielded a guanine-cytosine content in the range of 37–39%. The length range of the sequenced reads was 35–151 bp. Sample ONTGs-5, sequenced by MinION (ONT), had 2.19 Gb of bases passing the sequencer's inner filters and an estimated sixfold coverage of the predicted genome size. The size length range was 89–55,853 bp. The percentage of guanine-cytosine content was 35%.

Following pre-processing of the Illumina NextSeq samples with the fastp program, the percentage of guanine-cytosine content was found to be identical to that observed before processing, at 37%. The sequence length range was 100–151 bp, resulting in fewer reads (Additional file 2: Table S1). The processed Illumina NovaSeq reads exhibited a guanine-cytosine content of 37–38%. Following preprocessing, the total numbers of reads were 58 million and 253 million (Additional file 2: Table S1).

### Assembly evaluation

Using only Illumina-NextSeq short-read data, different assemblers generated distinct draft assemblies (Table 1).

**Table 1:** Metrics of the Illumina-NextSeq sample assembly drafts from a selection of QUAST and BUSCO data

Sample	Assembler	Contigs	Total length (pb)	N50 (pb)	GC (%)	BUSCO*(%)
ILGs-1	SPAdes	73,799	254,431,934	6747	37.91	54.1
ILGs-1	SoapDeNovo2	35,667	25,836,549	687	38.87	1.2
ILGs-1	Platanus	52,833	84,533,917	2118	38.6	20.6
ILGs-1	SGA	104,106	77,372,582	695	38.87	2.7
ILGs-1	AbySS	155,591	138,089,355	851	38.48	8.3
ILGs-1	Unicycler	180,012	257,638,835	1752	38	19.3
ILGs-2	SGA	36,935	24,302,369	624	38.57	0.6
ILGs-2	SPAdes	188,774	320,734,975	2477	38.02	31,1
ILGs-2	SoapDeNovo2	31,720	23,225,428	691	38.9	1.2

^*^Complete BUSCO genome mode

The SPAdes assembler was inferred to be the best, as evidenced by the higher N50 value observed in sample ILGs-1 (6457) and sample ILGs-2 (2477). Furthermore, the SPAdes assembly yielded the most accurate BUSCO results, with 54.1% observed in sample ILGs-1 and 31.1% in sample ILGs-2.

Next, only Illumina-NovaSeq short-read data was assembled using SPAdes (Table 2).

**Table 2:** Metrics of draft assemblies obtained with SPAdes and Illumina sequencing samples

Sample	Sequencer	Contigs	Total length (pb)	N50 (pb)	GC (%)	BUSCO*(%)
ILGs-3 SPAdes	NovaSeq	72,841	225,114,680	5733	37.5	50.2
ILGs-4 SPAdes	NovaSeq	120,494	316,541,976	5442	37.62	54.8
ILGs-1 SPAdes	NextSeq	73,799	254,431,934	6747	37.91	54.1
ILGs-2 SPAdes	NextSeq	188,774	320,734,975	2477	38.02	31.1

^*^Complete BUSCO genome mode, nematoda db

#### Hybrid approach

The optimal assemblies for the read and long-read sequencing using the SPAdes assembler in the hybrid mode (based on short reads) were sample ILGs-3 (Illumina NovaSeq) and sample ONTGs-5 (ONT) with the fewest contigs assembled (72,960), the highest N50 value (9834), the highest percentage of single BUSCOs (58.7%) (Additional file 2: Table S2), and the lowest percentage of duplicated, fragmented, and missing BUSCO genes (Additional file 2: Table S3). The hybrid approach improved the metrics of the individual assemblies of both short- and long-read-based assemblies (Additional file 2: Table S2). In the long-read-based approach, the best result is obtained for samples ONTGs-5 and ILGs-1 with Flye, Bowtie2, Samtools, Pilon, and Picard, although the metrics of both BUSCO and QUAST are similar between the different assemblies.

The samples with the best statistics were ILGs-3 and ILGs-1 (Illumina) with the SPAdes assembler. Combined with the long-read sample, (ONTGs-5, ONT) improves the assembly metrics, as does using the untrusted parameter with assemblies from the hybrid approach (combination of long and short reads, based on long reads) and subsequent hardening with other programs. A summary of assembly metrics is provided in Additional file 2: Figures S1 and S2.

The draft ILGs-3/ONTGs-5 SPAdes hybrid Pilon/Picard ONTGs-5/ILGs-1 assembly was referred to as version 1 (V1). After soft masking and purging, it was renamed version 1.2 (V1.2).

### Mitochondrial genome and structural and functional annotation

The published mitochondrial genome is 14,079 bp in size. A total of 17 scaffolds of the version assembly were aligned against the published mitochondrial genome, with almost 90% of the reference mitochondrial genome being assembled (Additional file 2: Figure S3).

The *G. spinigerum* V1.1 draft genome was 264.48 Mb in size (NCBI BioProject accession no. PRJNA1141240) and assembled into 70,253 scaffolds (N50: 11,416 bp). We assembled with RNAseq data; the number of genes was 27,592. Genome annotation produced 30,047 predicted protein-coding sequences. After purging repetitive elements and duplicates, there were 11,866 genes.

The *G. spinigerum* V1.2 draft genome was 222.26 Mb in size (NCBI BioProject accession no. PRJNA1141240) and assembled into 32,589 scaffolds (N50: 14,149 bp) (Tables 3, 4).

**Table 3:** Assembly draft metrics, after combining different assembly approaches, obtained with QUAST and BUSCO

Samples	Assembler	Contigs	Total length (pb)	N50 (pb)	GC (%)	BUSCO*(%)
ILGs-1 *Pilon/Picard ONTGs-5- ILGs-1*	SPAdes *Untrusted*	105,154	288,742,554	6608	37.77	58.1
ILGs-3 *Pilon/Picard ONTGs-5- ILGs-1*	SPAdes *Untrusted*	80,021	269,148,826	8645	37.67	58.3
ILGs-3 *Pilon ONTGs-5- ILGs-1*	SPAdes *Untrusted*	80,014	269,151,703	8646	37.67	59.1
ILGs-1 *Pilon ONTGs-5- ILGs-1*	SPAdes *Trusted*	100,998	299,435,360	7492	37.8	57.5
(V1) ILGs-3—ONTGs-5 *Pilon/Picard ONTGs-5- ILGs-1*	SPAdes hybrid *Untrusted*	70,253	264,481,023	11,416	37.64	61.4
(V1.2) ILGs-3—ONTGs-5 *Pilon/Picard ONTGs-5- ILGs-1*	SPAdes hybrid *Untrusted* Soft-masked purged	32,589	222,261,784	14,149	37.34	69.6

^*^Complete BUSCO genome mode

**Table 4:** Summary features of genome assemblies for *Gnathostoma spinigerum*

Assembly version	Assembly size (Mb)	Scaffolds	N50	GC (%)	BUSCO genome (%)	Genes	BUSCO annotation (%)
v1.1	264.48	70,253	11,416	37.64	61.4	27,592	62.3
v1.2	222.26	32,589	14,149	37.34	69.6	11,866	62.6

**Fig. 4:**
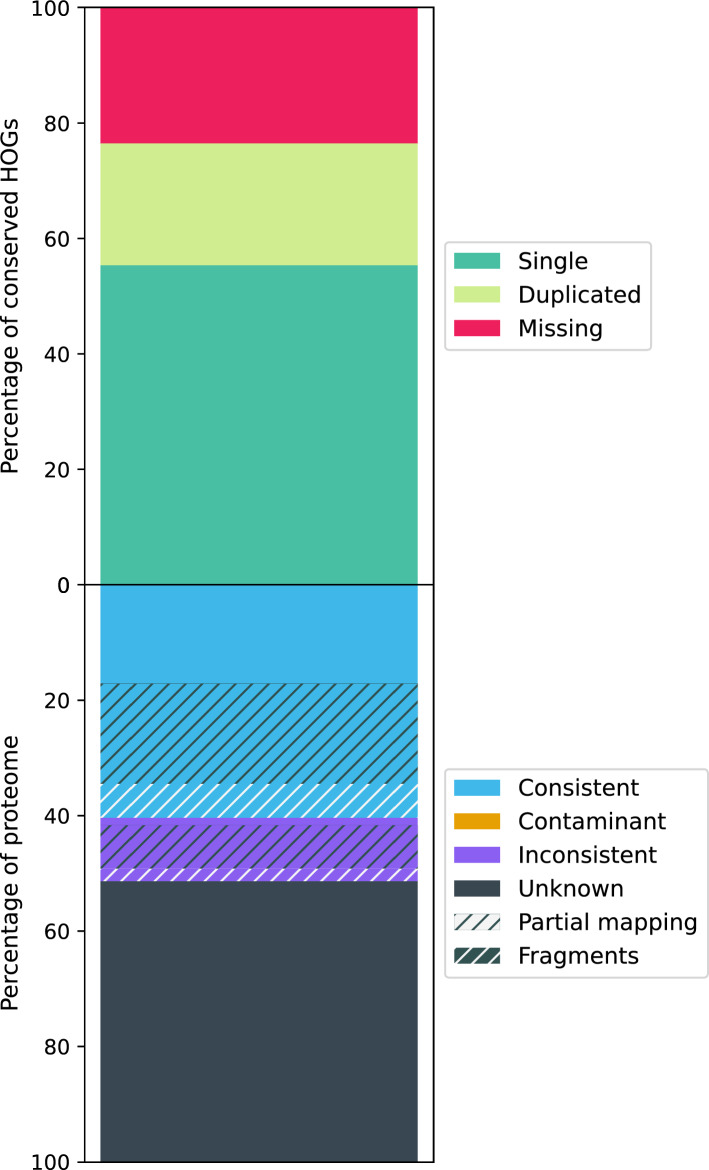
Percentage of proteome and conserved HOGs of *Gnathostoma spinigerum* annotated assembly. HOGs: Hierarchical Orthologous Groups. The graph categorized the HOGs as single, duplicated, or missing and the proteome as consistent, contaminant, inconsistent, unknown, partial mapping, and fragments

The number of proteins was 11,865, the total consistent lineage placement was 7249 (61.10%), and the number total contaminants was 0 (Table 5 and Fig. 4). The clade most consistent with the taxonomic distribution of gene families was Spirurina.

**Table 5:** Features of the genome of *Gnathostoma spinigerum* V1.2

Number of genes/mRNA	11,866
Gene length^a^	2786 ± 6238
mRNA length^a^	921 ± 841
Coding domain length^a^	921 ± 841
Number of exons	70,087
Exon length^a^	921 ± 841
Protein length^a^	307 ± 280
Completeness:	
Complete BUSCOs^b^	2,179 (69.6%)
Complete single-copy BUSCOs	2,141 (68.4%)
Complete and duplicated BUSCOs	38 (1.2%)
Fragmented BUSCOs	306 (9.8%)
Missing BUSCOs	646 (20.6%)
OMArk completeness (conserved HOGs):	
Single	3551 (67.27%)
Duplicated	390 (7.39%)
Missing	1338 (25.35%)
OMArk consistency^c^	
Total consistent lineage placements	7249 (61.10%)
Total inconsistent lineage placements	748 (6.30%)
Contaminants	0
Unknown	3868 (32.60%)

^a^Lengths (bp); mean ± standard deviation

^b^Number of BUSCOs identified (in genome mode) using nematoda_odb10 dataset (3131 genes)

^c^Proportion of annotated protein-coding genes in the metazoan proteome

The percentage of proteome and conserved HOGs of *G. spinigerum* annotated assembly is displayed in Fig. 4.

EggNOG-mapper annotated 11,071 (93.30%) predicted proteins; there was a predominance of genes involved in biological processes and cellular components. The most abundant COG categories were “Function unknown (S)” (22.7%), “Signal transduction mechanisms (T)” (10.7%), and “Post-translational, modification, protein turnover, chaperones (O)” (8.8%) (Table 6).

**Table 6:** Metrics of the annotated assembly with EggNOG

Metric	Value
Total proteins	11,071 (93.30%)
Annotated proteins	10,282 (92.00%)
Proteins with GO terms	7662 (74.00%)
Proteins with KEGG terms	7398 (71.00%)

^*^GO: Gene Ontology

Ten gene models containing the CAP domain (PF00188) were identified, representing the SCP/TAPS family in *G. spinigerum.*

Functional annotation of the *G. spinigerum* protein-coding set revealed a diverse repertoire of M10 matrix metalloproteinases (MMPs). Based on their domain architecture and comparative analysis with host and other nematode orthologs, these enzymes were classified into three distinct structural groups: one containing those with Signal Peptide, Propeptide, Catalytic, Hinge, and Hemopexin-like domains; another containing sequences with only Signal Peptide, Propeptide, and Catalytic domains; and finally, another group with a single sequence containing only an apparently incomplete catalytic domain (Table 7).

**Table 7:** Domain architecture and predicted localization of *Gnathostoma spinigerum* matrix metalloproteinases (MMPs)

Sequence ID (*G.spinigerum* genome)	Key domains present	Key features	MMP type	Secretion
G9182.T2	Signal peptide + propeptide + catalytic + Hinge + hemopexin-like	Resembles human MMP-2/9	Typical nematode MMP	Secreted
G1280.T1	Signal peptide + propeptide + catalytic + Hinge + hemopexin-like	Resembles human MMP-2/9	Typical nematode MMP	Secreted
G9438.T1	Signal peptide + propeptide + catalytic + Hinge + hemopexin-like	Resembles human MMP-2/9	Typical mematode MMP	Secreted
G12031.T2	Signal peptide + propeptide + catalytic	Similar to *Anisakis* matrilysins, lacks the hemopexin-like and the Hinge domain	MMP-7-like	Secreted
AAF82802	Signal peptide + propeptide + catalytic	Similar to *Anisakis* matrilysins, lacks the hemopexin-like and the Hinge domain	MMP-7-like	Secreted
G8944.T1	Signal peptide + propeptide + catalytic	Similar to *Anisakis* matrilysins, lacks the hemopexin-like and the hinge domain	MMP-7-like	Secreted
G7562.T1	Catalytic	Possible intracellular form or fragmented gene model	"Minimal" MMP	Non-secreted

### Computational simulation of the interaction between putative metalloproteinases M10 and tissue inhibitors of metalloproteinases (TIMPs)

**Fig. 5:**
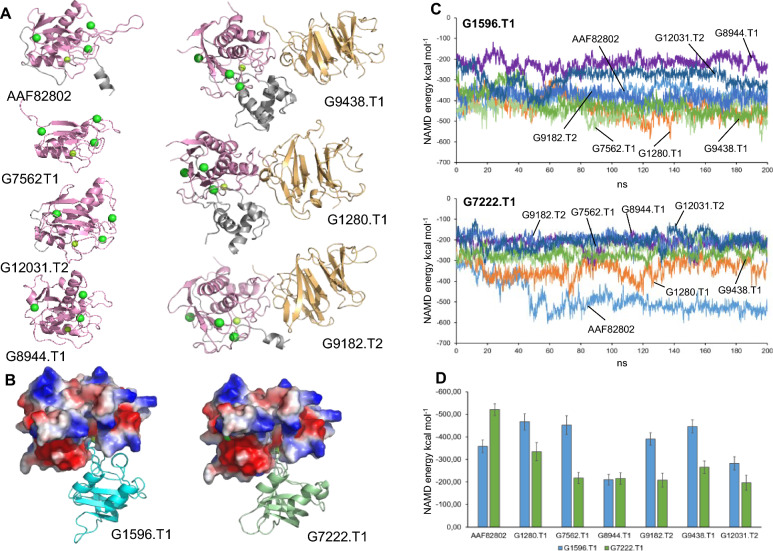
Structural models and computational analysis of the interaction between metalloproteinases M10 and TIMPs. **A** Structural models of the seven metalloproteinases M10 (AAF82802, G12031.T2, G9182.T2, G1280.T1, G9438.T1, G8944.T1, and G7562.T1). Catalytic domains are depicted in magenta. Putative positions of Ca +  + (green) and Zn +  + ions (light green) are indicated. **B** Structural model of the interaction between the catalytic domain of the metalloproteinase AAF82802 (showing the electrostatically charged surface) and the model of tissue inhibitors of metalloproteinases (TIMPs) G7222.T1 (left) and G1596.T1 (right), represented as secondary structures. **C** Interaction energy measured, using NAMD, over 200 ns of molecular dynamics between inhibitor G1596.T1 (upper panel) or G7222.T1 (lower panel) and the seven metalloproteinases M10. **D** Plot showing the mean ± SD values of energy shown in **B** and **C** and corresponding to the last 100 ns of the molecular dynamics trajectory. Blue bars: G1596.T1; green bars: G7222.T1

After identifying the sequences of six new M10 metalloproteinases (G12031.T2, G9182.T2, G1280.T1, G9438.T1, G8944.T1, and G7562.T1) and two tissue inhibitors of metalloproteinases (TIMPs) (G7222.T1 and G1596.T1), molecular modeling and molecular dynamics techniques were used to study the possible interactions between the M10 metalloproteinases and the TIMPs. The previously described M10 metalloproteinase (AAF82802) was also included in the computational simulation. First, structural models of the M10 metalloproteinases (Fig. 5A) and TIMPs (Fig. 5B) were generated. The M10 metalloproteinases can be divided into two groups based on the length of the genomic sequence. The sequences of the proteins AAF82802, G7562.T1, G12031.T2, and G8944.T1 mainly correspond to the catalytic domain (in magenta in Fig. 5A). The sequences of the proteins G9438.T1, G1280.T1, and G9182.T2 contain both the catalytic domain (in magenta in Fig. 5A) and the propeptide and the hemopexin domains (in gray and ochre, respectively, in Fig. 5A). The G7562.T1 protein does not appear to have a complete catalytic domain based on its sequence length. However, both possible Zn^2^⁺ and Ca^2^⁺ binding sites appear to be present. Moreover, after 200 ns of unrestricted molecular dynamics simulation, the overall structure of the domain did not change, suggesting that it may function as a catalytically active protein.

After generating the individual models, we generated models of possible interaction complexes between M10 metalloproteinases and TIMPs (for a total of 14 models), using the structure of a known homologous complex [40] as a template. Figure 5B illustrates the interaction model between the metalloproteinase AAF82802 and the two TIMPs. We then subjected these models to a 200-ns molecular dynamics simulation to evaluate the stability of each metalloproteinase's interaction with the two TIMPs. The interaction energy was obtained along the trajectories using NAMD [44], as shown in Fig. 5C. Finally, the energy values of each model during the first 100 ns of the simulation were discarded, assuming stabilization of the complexes during this time. The values from the remaining 100 ns were then averaged to obtain mean values ± SD (Fig. 5D).

The energy analysis appears to categorize the metalloproteinase-TIMP pairs into three groups. The first group consists of the metalloproteinases G8944.T1 and G12031.T2, which exhibit modest interaction energies between their active centers and both TIMPs. The second group consists of the metalloproteinases G1280.T1, G7562.T1, G9182.T2, and G9438.T1. This group shows a clear preference for TIMP G1596.T1 (blue bars in Fig. 5D) over TIMP G7222.T1 (green bars). The third group consists of a single member: the metalloproteinase AAF82802. This metalloproteinase shows the opposite behavior, with lower interaction energy (indicating higher affinity) with G7222.T1 than with G1596.T1.

## Discussion

The present study reports the first complete genome assembly and annotation of *G. spinigerum*, a nematode parasite that causes severe or even fatal eosinophilic meningitis in humans. The genome was sequenced, assembled, and annotated using a hybrid assembly approach and will be a valuable resource for the characterization of the genetic basis of the parasite and the identification of potential drug and diagnostic candidates.

A pool of larvae was employed instead of a single individual, as the requisite concentration could not be achieved with a single larva. For the Illumina sequencing, the number of individuals was 51 and 10 and with ONT; 162 larvae were utilized. Other de novo genome assemblies have used a pool of larvae with successful results, such as *Wuchereria bancrofti*, *Anisakis simplex*, *Enterobius vermicularis*, and *Brugia pahangi* [45]. However, it can reasonably be assumed that this factor contributes to elevated heterozygosity rates and a high number of SNPs. These factors—assembly fragmentation and high genetic diversity—likely influenced the total number of genes identified in our study. Because we sequenced a pool of many larvae, the assembly software may have occasionally struggled to distinguish between two versions of the same gene (alleles) and instead reported them as two separate genes. This can lead to an overestimation of the size of certain gene families. Conversely, because the assembly is still fragmented (as indicated by the 69.6% BUSCO score), some genes may be incomplete or missing from our data, which could lead to an underestimation of other gene groups. While these are common challenges when working with wild, non-inbred parasites, our current gene counts should be viewed as a preliminary draft. Future work using single-worm sequencing and higher-level scaffolding will be necessary to confirm the exact copy number of these genes. In the future, obtaining adult specimens would facilitate whole-genome sequencing from a single worm and permit the construction of a contact map using Hi-C technology to assemble contigs into chromosomes.

Mitochondrial genomes should be included in the evaluation of whole-genome assemblies, as they are indispensable components of an organism's complete genome [46]. In our case, despite the challenges of performing mitochondrial assembly without organelle-specific purification, the results obtained from the first assembly version were encouraging. Practically the entire mitochondrial genome was covered. This aspect is reinforced by the absence of sample contamination, whether from human, host (eel), bacterial, or viral organisms.

*Gnathostoma spinigerum* invades the central nervous system by migrating along the nerve roots. The larva has cuticular spines and apical rows of hooks, which allow it to burrow through soft tissues, resulting in necrosis in the brain and spinal cord and cerebral haemorrhages directly associated with its mortality [47, 48]. A hypothetical role in the degradation of extracellular matrix macromolecules of host tissues has been assigned to a 24-kDa secreted matrix metalloproteinase (MMP)-like protein from *G. spinigerum* L3 (AAF82802), identified as matrilysin [49]. Matrilysins have a simpler structure than other MMPs, characterized by the absence of a C-terminal hemopexin domain [50]. Besides, *Gnathostoma* L3 MMP has been proven to be antigenic and useful in human diagnosis [51, 52]. The immunogenic proteins of *G. spinigerum*, are known to be located in the 24-kDa region, and efforts have been made to isolate and characterize immunodiagnostic candidates and drug targets by proteomics and RNA seq experiments [52–54], always with the constraint of the absence of a reference genome. It is known that inflammatory and parasitic MMPs may be suitable therapeutic targets to prevent blood-brain barrier disruption [55]. Indeed, one of the most abundant metalloproteinase inhibitors expressed in *G. spinigerum*, homologous to the putative *Caenorhabditis elegans* inhibitor cri-2, for which no sequence has been identified so far, has been postulated as a potential target for both anthelmintics and vaccines [54]. In this work, we have identified six new M10 metalloproteinases and two tissue inhibitors of metalloproteinases (TIMPs). Our analysis revealed that these MMPs possess two distinct architectures: "short-form" matrilysin-like enzymes and "long-form" typical nematode MMPs. As previously described, the matrilysin-like proteins (e.g. AAF82802 and G12031.T2) lack the C-terminal hemopexin-like domain, a feature shared with invasive stages of *Anisakis simplex*. Their simplified structure likely facilitates rapid diffusion through host tissues during the larva’s characteristic burrowing migration. Conversely, the typical MMPs (e.g. G9182.T2) contain a hemopexin domain, resembling human MMP-2/9 (gelatinases). These "long-form" MMPs are common in other clade III and IV nematodes like *Strongyloides*, in which they are used to target the degradation of host collagen and basement membranes. The presence of signal peptides in nearly all identified MMPs confirms their role as secreted factors that directly facilitate the necrosis and hemorrhaging observed along larval migration pathways in the central nervous system[55].

The computational analysis of interactions homologous to the putative *C. elegans* inhibitor cri-2 between these new M10 metalloproteinases, the one previously described, and the two TIMPs detected in the genome, suggests different behaviors for each. This relatively low selectivity for different metalloproteinases shown by TIMP G1596.T1 is frequent in TIMPs, usually forming tight 1:1 complexes and also participating in pro-MMP activation and in suppression of different biological functions, such as tumor growth, matrix binding, inhibition of angiogenesis, and induction of apoptosis [56]. The metalloproteinases G8944.T1 and G12031.T2, the latter a matrilysin structurally similar to AAF82802, are not predicted to exhibit strong affinity for either TIMP. This could imply a distinct mode of action, the presence of additional TIMPs in the genome that could not be detected in this first draft, or the existence of other proteins with equivalent functions. Future comprehensive studies could reveal the utility of the identified TIMPs as therapeutic targets as well as the potential role of novel MMPs in pathogenesis or diagnosis. In addition to matrilysins, we performed a survey of other gene families classically associated with nematode parasitism. Specifically, we identified 10 members of the SCP/TAPS family (Pfam: PF00188) within the *G. spinigerum* genome. These proteins, frequently identified in the secretomes of parasitic nematodes, are implicated in the transition to parasitism, larval migration, and the modulation of the host immune response. While the identification of 10 candidates highlights a significant repertoire of these virulence-associated factors, this count should be considered a preliminary survey. Given the high heterozygosity and fragmented nature of the current draft assembly, future work is needed to distinguish between true paralogous expansions and uncollapsed allelic variants.

The application of next-generation sequencing (NGS) techniques to the diagnosis of infectious viral, bacterial, and parasite meningitis and eosinophilic encephalitis in a single step, using cerebrospinal fluid as a sample, has been the subject of recent reports in the scientific literature. One such study, conducted across eight hospitals in the US, found the presence of *Angiostrongylus cantonensis* in two cases [57]. This parasite, along with *G. spinigerum*, is the primary etiological agent responsible for parasitic eosinophilic meningitis. Although the incidence of *A. cantonensis* meningoencephalitis is higher than that of *G. spinigerum*, cases of neurognathostomiosis are more severe, with more sequelae and higher mortality (< 1% versus 7–25%) [47, 58]. The availability of the *G. spinigerum* genome would enable detection of the worm in these diagnostic/analysis panels. For example, in regions endemic to parasitic eosinophilic meningitis, such as Southeast Asia, the availability of the *A. cantonensis* genome has already facilitated the diagnosis of numerous cases, which are particularly relevant in the pediatric population because of the immaturity of their immune systems [57, 59, 60]. The same could be applied to cerebral *Gnathostoma* cases, where early diagnosis is essential because of the severity of the pathology and sequelae [61, 62]. Continued development of genomic technologies will reduce the costs of sequencing and library preparation, enabling the routine genomic sequencing of clinical samples. However, this approach requires a genome, such as the one generated in this article.

## Conclusions

The first genome assembly of *G. spinigerum* marks a major advance in parasitic genomics, yet this study represents only an initial step toward full genomic characterization. To resolve structural and functional complexities, future work should prioritize high-resolution sequencing of a single individual. Chromosome-scale assembly will further improve accuracy, enabling deeper insights into the parasite’s genetic architecture. Despite current limitations, this genome provides a critical resource for advancing diagnostics, drug discovery, and basic research on this species. In addition, we present novel molecules that could lead to new research opportunities regarding their potential role in pathogenesis/diagnosis or as a therapeutic target for MMP and TIMPs, respectively.

## Supplementary Information

Additional file 1.

Additional file 2.

## Data Availability

Following the FAIR principles, all datasets generated and analyzed during the *Gnathostoma spinigerum* Genome Project are openly available at the following addresses: BioProject PRJNA1141240 *Gnathostoma spinigerum* isolate:aL3 Genome sequencing.https://www.ncbi.nlm.nih.gov/datasets/genome/GCA_043882145.1

## Abbreviations

BUSCO: Benchmarking universal single-copy orthologs
GC: Guanine cytosine
HPC: High-performance computing cluster
IL: Illumina, short-read sequencing
L3: Third-stage larvae
MMP: Matrix metalloproteases
N50: Sequence length of the shortest contig at 50% of the total assembly length
ONT: Oxford Nanopore Technologies
bp: Base pair
PCR: Conventional polymerase chain reaction
QUAST: Quality Assessment Tool for Genome Assemblies
TIMPs: Tissue inhibitors of metalloproteinases
