# Supplementary material for: De novo genome assembly and annotation of Gnathostoma spinigerum
Source: Parasit Vectors. 2026 Apr 11;19:223. doi: 10.1186/s13071-026-07378-1 (PMC13185349; doi:10.1186/s13071-026-07378-1)
Supplement: Supplementary file 1 — Additional file 1. [file 13071_2026_7378_MOESM1_ESM.docx]

**Supplementary File 1: Text 1.** Bioinformatics software, assemblers procedures used and additional analysis

**Bioinformatics software:**

The high-performance computing (HPC) cluster has the Linux CentOS-8 (4.18.0-305.19.1el8_4.x86_64) operating system installed. The main nodes operated were Ivy Bridge (Xeon ES-2670v2) with 16 nodes and a RAM memory of 256 GB and Cascade Lake (Xeon Gold 6242) with 16 nodes and RAM memory of 384 GB, 512 GB and 2,048 GB.

Preprocessing was conducted using the fastp program (v.0.20.0), with the following parameters set: 1) A minimum read size of 100 bp (length_required=100), 2) Minimum Phred quality score of 15 (qualified_quality_phred=15), 3) Trimming of regions from the 3' end with an average quality below 15 (cut_mean_quality=15, cut_tail), 4) Trimming of homopolymeric regions at the 3' end (--trim_poly_x), 5) Removal of sequences corresponding to adapters (included by default).

**Assemblers used and procedures:**

The SGA assembler outlines a three-step process for assembly: error correction, contig assembly and scaffolding. In the first step, the kmer value (CK = 41) was selected as 41. Other parameters included a minimum kmer coverage of 2 (COV_FILTER=2) for filtering, an overlap parameter of 55 (MOL=55) for read fusion, and an overlap parameter of 75 (OL=75) for the final assembly. The SPAdes program was employed, utilising the options "-careful" and without this parameter, in addition to the values of kmer 41, 63, 81, 107 and 127.

For SoapDeNovo2, the parameters set were as follows: maximum read size (max_rd_len=150), average insert size (avg_ins=223), contig and scaffold-level assembly, and minimum contig alignment length (map_len=32). The kmer values were 51, 63, 127 and 143. The draft assembly was performed using Platanus in two steps: the first was contig assembly, the second was scaffolding. The parameters selected for scaffolding were mean insert size (a1=232) and standard deviation of insert size (d1=56). The ABySS assembler was employed with the following parameters: paired-end mode, 30 MPI processes (np=30), a kmer size value of 107 (k=107), and 5 bloom filter mode (B=5G). The default parameters of the Unicycler program were employed in this mode, with kmer values of 27, 53, 71, 99 and 111 selected.

The Flye assembler (v.2.9) was run using three modalities, resulting in three distinct assemblies. The first assembly was created with raw reads, without any correction, using the nano-raw parameter. The second assembly was created with corrected reads, processed using the nano-raw parameter. The third assembly was created with raw reads, indicating an estimated genome size, using the nano-raw parameter and a genome size of 260 Mb (genome-size=260m). Raw reads were indicated, with an estimated genome size of 260 Mb (genome-size=260m), using the nano-raw parameter.

The NECAT program (v.0.0.1) was executed by performing the steps indicated by the developers: necat correct, necat assemble and necat bridge. The main parameters indicated a minimum read size of 500 bp and a minimum coverage of 20.

QUAST was executed with the default parameters, and the following main metrics were reported: number of contigs, N50, total assembly length, N75, L50, L75, and guanine-cytosine percentage (GC (%)). For the analysis, all statistics were based on contigs larger than 500 bp. Furthermore, BUSCO (version 5.3.2) was initiated using the euk-genome-met mode, with the metaeuk gene predictor, and the nematode database (nematoda_db), which was created in August 2020 and comprises seven genomes and 3,131 BUSCOs.

The SPAdes assembler operated in hybrid mode using k-mer values of 63, 81, 107, and 127. Various optional parameters such as --nanopore, --untrusted-contigs, and --careful were incorporated, with different combinations tested, some omitting certain optional parameters. Specifically, the --untrusted-contigs parameter utilized each draft assembly obtained from sequencing samples other than the one being analyzed. Bowtie2 software (v.2.4.2) was employed to build an index of the ONT read assembly using bowtie2-build in --large-index mode. Alignment of FASTQ files containing short reads against the ONT read assembly was then performed using Bowtie2. SAMtools package (v.1.12) was utilized to convert alignment results into BAM format (samtools view -Sb), sort them (samtools sort), and generate indices (samtools index). Pilon program (v.1.23) was executed with default parameters, specifying the Flye assembly derived from ONT readings using the -genome option to enhance assembly quality by correcting inconsistencies with the generated reads. Picard software (v.2.25.1) was employed with the MarkDuplicatesWithMateCigar parameter enabled to remove duplicate reads (REMOVE_DUPLICATES=true) from the processed data.

**Additional analysis:**

Pre-assembly contamination screening (Kraken2): The Kraken2 program [34] (v.2.1.2) was employed to ensure the absence of contamination in short read sequences from other species. The primary concern was potential contamination from eel (*M. albus*), the intermediate host from which larvae were extracted. Additionally, to identify and eliminate other potential sources of contamination, databases containing bacterial, archaeal, viral, and human sequences from the Minikraken v2 database (available at https://benlangmead.github.io/aws-indexes/k2) were queried against the sequences obtained from Illumina technology sequencing of the samples. The results were visualized using KronaTools [35] (v.2.8.1) to generate graphical representations for further analysis and verification.

Post-assembly proteome validation (OMark): To confirm the taxonomic identity of the final gene set, we used OMark software, a genome-scale tool that assesses proteome integrity based on orthology. The OMark analysis reported 0% contamination, and the taxonomic assignment was highly consistent with the Nematoda/Spirurida lineage. This genome-scale evidence complements our microscopy and COI barcoding, confirming that the assembly represents a clean, nuclear genome of *G. spinigerum*.
