# Supplementary material for: De novo genome assembly and annotation of Gnathostoma spinigerum
Source: Parasit Vectors. 2026 Apr 11;19:223. doi: 10.1186/s13071-026-07378-1 (PMC13185349; doi:10.1186/s13071-026-07378-1)
Supplement: Supplementary file 2 — Additional file 2. [file 13071_2026_7378_MOESM2_ESM.docx]

**Additional File 2:**

**Table S1.** Sequencing results pre- and post-processing on the two platforms.

| **Sample** | **Platform** | **Sequencer** | **Library** | **Raw reads** | **Processed reads** |
| --- | --- | --- | --- | --- | --- |
| ILGs-1 | Illumina | NextSeq | Nextera  DNA Flex | 143,645,727 | 85,416,369 |
| ILGs-2 | Illumina | NextSeq | Nextera  DNA Flex | 347,533,174 | 164,176,444 |
| ILGs-3 | Illumina | NovaSeq | DNA PCR-Free | 74,552,910 | 58,166,056 |
| ILGs-4 | Illumina | NovaSeq | DNA PCR-Free | 323,025,557 | 253,928,440 |
| ONTGs-5 | ONT | MinION | SQK-LSK110 | 2,178,642 | 1,731,866 |

**Table S2.** Metrics, of the draft assemblies hybrid approach, with different software, obtained using QUAST and BUSCO.

| **Samples** | **Assembler** | **Contigs** | **Total length** (pb) | **N50** (pb) | **GC** (%) | **BUSCO***(%) |
| --- | --- | --- | --- | --- | --- | --- |
| **Hybrid approach (based on short reads)** | | | | | | |
| ILGs-1 /ONTGs-5 | SPAdes hyb | 98,961 | 280,179,417 | 6,919 | 37.83 | 57.4 |
| ILGs-2 / ONTGs-5 | SPAdes hyb | 122,834 | 307,278,749 | 6,084 | 37.92 | 57.2 |
| ILGs-3 /ONTGs-5 | SPAdes hyb | 72,960 | 262,129,574 | 9,834 | 37.71 | 59.9 |
| ILGs-4 / ONTGs-5 | SPAdes hyb | 120,847 | 317,915,880 | 4,929 | 37.57 | 54.7 |
| ILGs-1/ ILGs-3/ ILGs-4/ ONTGs-5 | SPAdes hyb | 264,035 | 374,321,587 | 1,907 | 37.71 | 35.8 |
| **Combination of different approaches with untrusted-contigs parameter** | | | | | | |
| ILGs-4  *ILGs-1 /* *ONTGs-5* | SPAdes *Untrusted SPAdes* | 114,178 | 281,458,871 | 4,650 | 37.56 | 50.5 |
| ILGs-3  *ONTGs-5* | SPAdes *Untrusted Flye* | 74,058 | 263,961,864 | 9,910 | 37.66 | 60 |
| ILGs-4  *ONTGs-5* | SPAdes *Untrusted Flye* | 207,499 | 331,298,066 | 2,174 | 37.63 | 28.5 |
| ILGs-3 - ONTGs-5  *ILGs-1 /* *ONTGs-5* | SPAdes hyb *Untrusted SPAdes* | 82,139 | 272,400,619 | 8,603 | 37.74 | 58.7 |
| ILGs-1 - ONTGs-5  *ILGs-3 /* *ONTGs-5* | SPAdes hyb *Untrusted SPAdes* | 97,537 | 287,764,672 | 8,299 | 37.81 | 60.1 |
| **Hybrid approach (based on long reads)** | | | | | | |
| ONTGs-5  ILGs-3 | Flye  Bowtie2 Pilon | 16,806 | 154,906,995 | 12,962 | 37.44 | 38.4 |
| ONTGs-5  ILGs-1 | Flye  Bowtie2 Pilon | 16,806 | 154,890,617 | 12,953 | 37.45 | 38.6 |
| ONTGs-5  ILGs-3 | Flye  Bowtie2 Pilon Picard | 16,806 | 154,911,257 | 12,962 | 37.44 | 38.4 |
| ONTGs-5  ILGs-1 | Flye  Bowtie2 Pilon Picard | 16,806 | 154,891,890 | 12,954 | 37.45 | 38.9 |

*Complete BUSCOs genome mode. Note: The samples used to obtain the draft assembly specified by the untrusted-contigs parameter are shown in italics.

**Table S3.** Busco results of assemblies for *Gnathostoma spinigerum*

|  |  | | | | |  |  |  |  |  |
| --- | --- | --- | --- | --- | --- | --- | --- | --- | --- | --- |
| **Assembly version** | **BUSCO Results** | | | | | | | | | |
|  | **Complete** | | **C-single-copy*** | | **C-Duplicated*** | | **Fragmented** | | **Missing** | |
|  | **%** | **n** | **%** | **n** | **%** | **n** | **%** | **n** | **%** | **n** |
| ILGs-1 *Pilon/Picard ONTGs-5-ILGs-1* | 58.10 | 1,819 | 56.8 | 1,777 | 1.3 | 42 | 7.9 | 247 | 34.0 | 1,065 |
| ILGs-3 *Pilon/Picard ONTGs-5-ILGs-1* | 58.30 | 1,827 | 57.1 | 1,788 | 1.2 | 39 | 8.3 | 261 | 33.4 | 1,043 |
| I LGs-3 *Pilon ONTGs-5-ILGs-1* | 59.10 | 1,849 | 57.8 | 1,809 | 1.3 | 40 | 7.9 | 247 | 33.0 | 1,035 |
| ILGs-1 *Pilon ONTGs-5-ILGs-1* | 57.50 | 1,802 | 55.5 | 1,738 | 2.0 | 64 | 8.1 | 255 | 34.4 | 1,074 |
| ILGs-3 ONTGs-5 *Pilon/Picard ONTGs-5-ILGs-1* | 61.40 | 1,924 | 60.0 | 1,879 | 1.4 | 45 | 7.8 | 145 | 30.8 | 962 |
| ONTGs-5 ILGs-3 | 38.40 | 1,205 | 37.9 | 1,188 | 0.5 | 17 | 6.5 | 204 | 55.1 | 1,722 |
| Flye Bowtie2 Pilon |  |  |  |  |  |  |  |  |  |  |
| ONTGs-5 ILGs-1 | 38.60 | 1209 | 38.0 | 1.19 | 0.6 | 19 | 6.6 | 207 | 54.8 | 1,715 |
| Flye Bowtie2 Pilon |  |  |  |  |  |  |  |  |  |  |
| ONTGs-5 ILGs-3 | 38.80 | 1,215 | 38.2 | 1,196 | 0.6 | 19 | 6.4 | 201 | 54.8 | 1,715 |
| Flye Bowtie2 Pilon Picard |  |  |  |  |  |  |  |  |  |  |
| ONTGs-5 ILGs-1 | 38.90 | 1217 | 38.2 | 1195 | 0.7 | 22 | 6.6 | 206 | 54.5 | 1,708 |
| Flye Bowtie2 Pilon Picard |  |  |  |  |  |  |  |  |  |  |
| ILGs-4 *ILGs-1* */ONTGs-5* | 50.5 | 1,581 | 49.2 | 1.54 | 1.3 | 41 | 8.6 | 269 | 40.9 | 1,281 |
| ILGs-3 *ONTGs-5* | 60 | 1,879 | 58.6 | 1,834 | 1.4 | 45 | 8 | 252 | 32 | 1 |
| ILGs-4 *ONTGs-5* | 28.5 | 892 | 27 | 845 | 1.5 | 47 | 8.6 | 268 | 62.9 | 1,971 |
| ILGs-3 ONTGs-5 | 58.7 | 1,836 | 57.4 | 1,796 | 1.3 | 40 | 8 | 251 | 33.3 | 1,044 |
| *ILGs-1 / ONTGs-5* |  |  |  |  |  |  |  |  |  |  |
| ILGs-1 ONTGs-5 | 60.1 | 1,883 | 58.7 | 1,838 | 1.4 | 45 | 7.8 | 243 | 32.1 | 1.005 |
| *ILGs-3/ONTGs-5* |  |  |  |  |  |  |  |  |  |  |

*Complete BUSCO genome mode, nematode database.


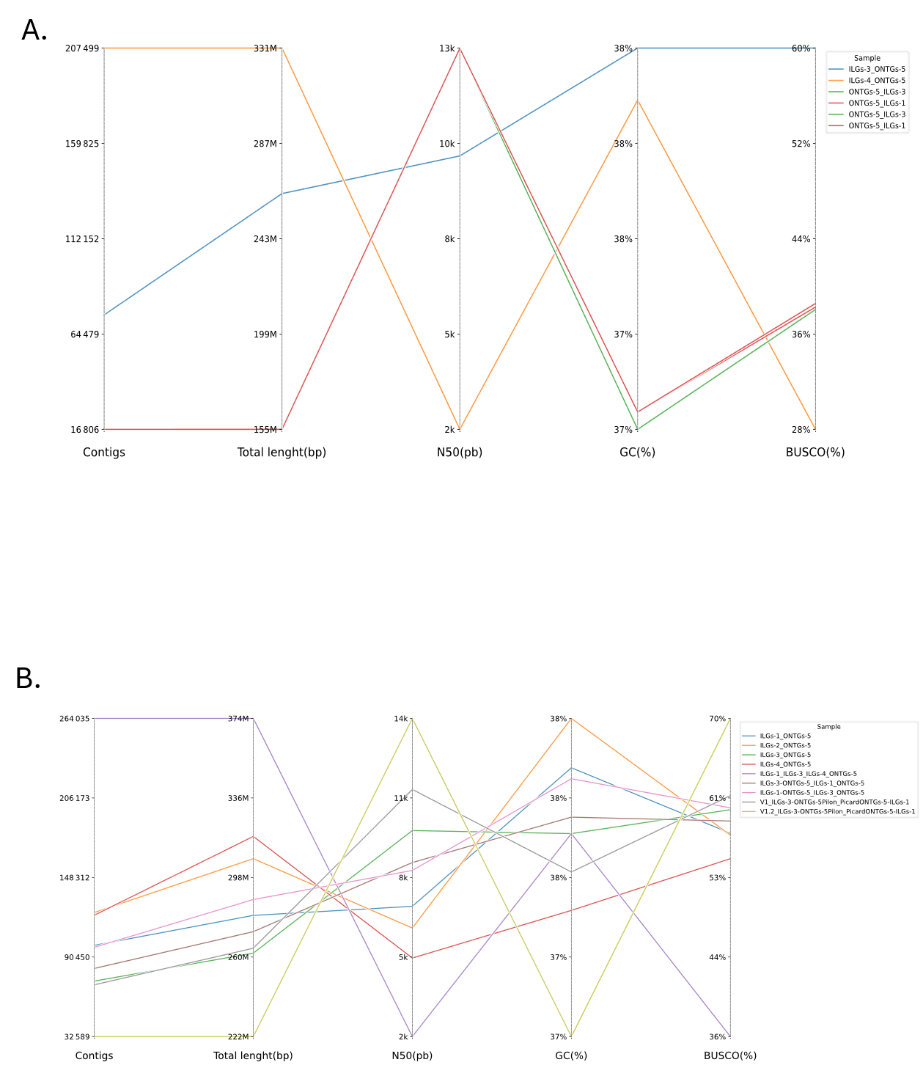


**Figure S1.** Parallel chart of hybrid assemblies metrics. **A**) Hybrid assemblies metrics (SPAdes and ONT). **B)** Different hybrid assemblies approaches metrics.


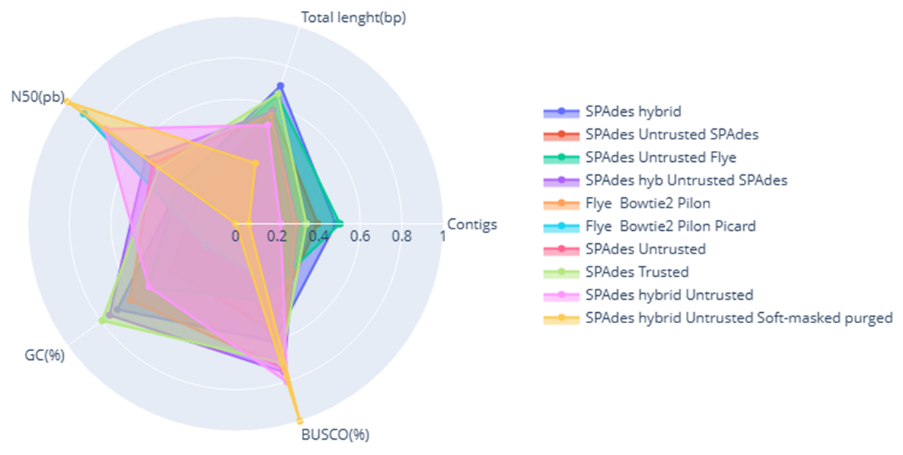


**Figure S2.** Spider chart of hybrid assemblies metrics. **GC**: guanine cytosine content. **bp:** base pair. Complete BUSCO genome mode, nematode database.

**Mitochondrial assembly**


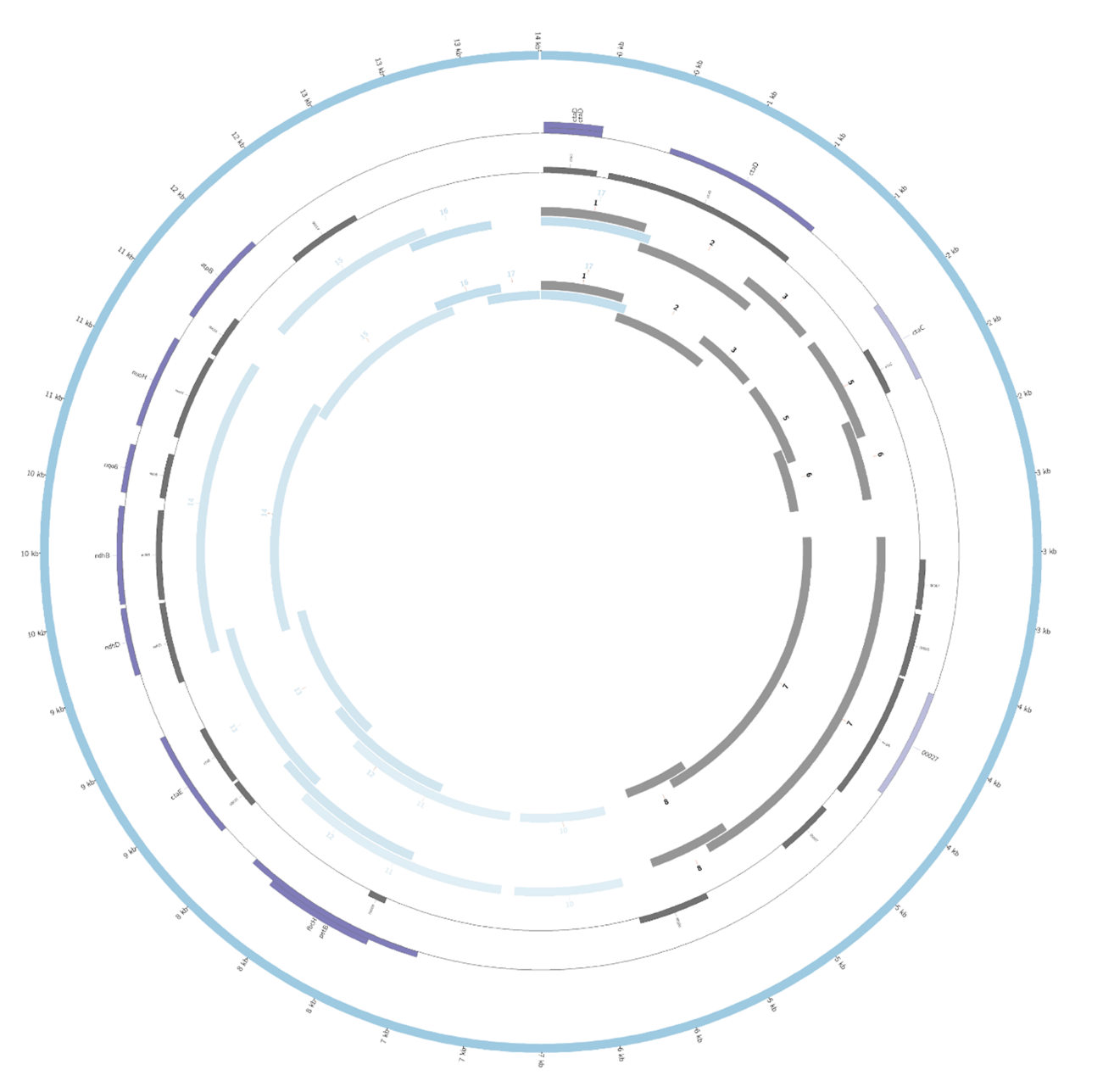


**Figure S3. Circos plot of the alignment of the v1.1 assembly with the reference mitochondrial genome.** The outer blue line represents the reference mitochondrial genome. The second and third layers are the results of automatic annotation with the sample (the draft assembly obtained, second layer) and the reference mitochondrial genome (third layer). The fourth and fifth layers represent two options of local alignments of the sequences of the scaffolds of our assembly with the mitochondrial genome. The numbers (1,2,3...) represent those selected scaffolds whose percentage of coverage is higher than 47.
